# A Novel Schiff Base of 3-acetyl-4-hydroxy-6-methyl-(2H)pyran-2-one and 2,2'-(ethylenedioxy)diethylamine as Potential Corrosion Inhibitor for Mild Steel in Acidic Medium

**DOI:** 10.3390/ma8062918

**Published:** 2015-05-26

**Authors:** Jonnie N. Asegbeloyin, Paul M. Ejikeme, Lukman O. Olasunkanmi, Abolanle S. Adekunle, Eno E. Ebenso

**Affiliations:** 1 Department of Pure and Industrial Chemistry, University of Nigeria, Nsukka 40001, Enugu State, Nigeria; E-Mails: niyi.asegbeloyin@unn.edu.ng (J.N.A.); paul.ejikeme@unn.edu.ng (P.M.E.); 2 Material Science Innovation and Modelling (MaSIM) Research Focus Area, Faculty of Agriculture, Science and Technology, North-West University (Mafikeng Campus) Private Bag X2046, Mmabatho 2735, South Africa; E-Mails: waleolasunkanmi@gmail.com (L.O.O.); sadekpreto@gmail.com (A.S.A.); 3 Department of Chemistry, Faculty of Science, Obafemi Awolowo University, Ile-Ife 220005, Nigeria

**Keywords:** Schiff base, electrochemical techniques, mild steel, adsorption, quantum chemical calculations

## Abstract

The corrosion inhibition activity of a newly synthesized Schiff base (SB) from 3-acetyl-4-hydroxy-6-methyl-(2H)-pyran-2-one and 2,2'-(ethylenedioxy)diethylamine was investigated on the corrosion of mild steel in 1 M HCl solution using potentiodynamic polarization and electrochemical impedance spectroscopic techniques. Ultraviolet-visible (UV-vis) and Raman spectroscopic techniques were used to study the chemical interactions between SB and mild steel surface. SB was found to be a relatively good inhibitor of mild steel corrosion in 1 M HCl. The inhibition efficiency increases with increase in concentration of SB. The inhibition activity of SB was ascribed to its adsorption onto mild steel surface, through physisorption and chemisorption, and described by the Langmuir adsorption model. Quantum chemical calculations indicated the presence of atomic sites with potential nucleophilic and electrophilic characteristics with which SB can establish electronic interactions with the charged mild steel surface.

## 1. Introduction

Mild steel is used in many industrial and structural applications due to its good mechanical strength and relatively low cost [1,2]. Acidic solutions commonly used in many industrial activities, including the steelmaking finishing process, constitute unfriendly corrosive media for mild steel [3]. The use of organic corrosion inhibitors has been identified as one of the most economical ways of reducing corrosion rate and protecting steel-made industrial facilities against corrosion [3,4]. The ability of Schiff base ligands to form stable complexes closely packed in the coordination sphere of metal ion introduces another class of compounds for corrosion inhibition [5]. Schiff bases are adsorbed on metal surfaces due to the presence of >C=N– groups [6]. This adsorption behavior leads to spontaneous formation of a monolayer covering the metal surface, consequently acting as effective corrosion inhibitor.

3-acetyl-4-hydroxy-6-methyl-(2H) pyran-2-one, commonly referred to as dehydroacetic acid, and its derivatives have been of research interest because of their interesting coordination chemistry, pharmaceutical importance and biological activities [7,8,9,10,11]. DNA binding and antibacterial screening of dehydroacetic acid complexes of Ru(II) and Ru(III) containing PPh_3_/AsPh_3_ have been recently reported by Chitrapriya *et al.* [12]. Dehydroacetic acid is well known for its fungicidal [13], herbicidal and antimicrobial activities [10]. It is also widely used in food technology, as a vitamin C stabilizer and as a preservative in food products like fish sausages [14]. Therefore, dehydroacetic acid and possibly its Schiff bases, are non-toxic and eco-friendly. The presence of O and N heteroatoms as well as >C=N– and >C=O functional groups in dehydroacetic acid/2,2'-(ethylenedioxy)diethylamine Schiff base may facilitate electronic interactions with a mild steel surface, leading to adsorption on the steel surface and consequently, inhibition of steel corrosion.

The present work is in furtherance of the continual search for eco-friendly, easy to synthesize and effective corrosion inhibitors. In this work, a novel Schiff base (SB) was synthesized based on condensation of 3-acetyl-4-hydroxy-6-methyl-(2H)pyran-2-one and 2,2'-(ethylenedioxy)diethylamine and investigated for its corrosion inhibition activities using electrochemical methods, spectroscopic techniques and quantum chemical calculations.

## 2. Results and Discussion

### 2.1. Synthesis of SB

The results of IR, NMR and elemental analyses confirmed successful synthesis of SB with the chemical structure (SB) shown under the experimental Section 3.3. The percentage yield and melting point of the whitish compound (C_22_H_28_N_2_O_8_) synthesized were 85% and 155 °C, respectively. The infrared, proton and carbon-13 NMR spectroscopic results of the product are given in Table 1. Detail assignments of these spectroscopic data have been described somewhere else [15].

**Table 1:** IR, ^1^HNMR, ^13^CNMR spectroscopy data and elemental analysis of SB.

IR (KBr cm^−1^)	1665 (C=N), 3454 (OH), 1703 (C=O),1254 (C-O), 1358 (C-N)	
^1^H NMR (CDCl_3_, δ, ppm)	2.11 (s, 3H, -C=C-CH_3_); 2.50 (s, 3H, N=C-CH_3_); 5.62 (s, 1H, CH_3_=C-H); 3.27δ (s, 4H); 3.70δ (m, 6H); 13.80δ (s, 1H, enolic OH)	
^13^C NMR (CDCl_3_, δ, ppm)	18.27, 19.58 (-CH_3_), 183.66 (O-C=O); 176.03 (C-OH); 162.47 (H_3_C-C-O); 107.49 (C=N); 68.52 (-CH_2_-O); 44.12 (CH_2_ –N)	
Elemental analysis	Experimental	C=58.32; H=6.24; N=6.25
	Calculated	C=58.92; H=5.98; N=6.44

### 2.2. Electrochemical Measurements

#### 2.2.1. Open Circuit Potential (OCP)

**Figure 1:**
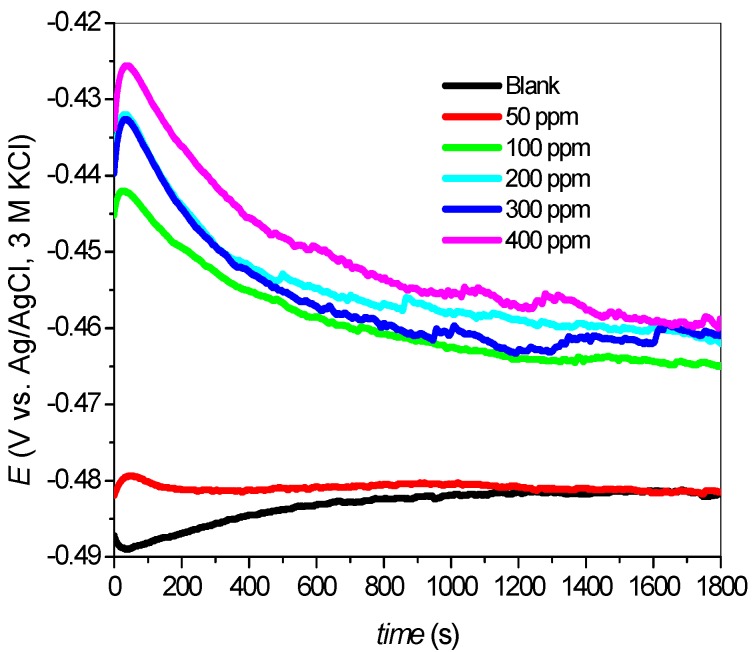
OCP scan for mild steel corrosion in 1 M HCl with and without various concentrations of SB.

The results of OCP measurements on mild steel corrosion in 1 M HCl with and without various concentrations of SB are as shown in Figure 1. A relatively steady potential (∆*E* = ±1 mV) was reached at about 1000 s of immersion in all cases. There was initial decrease in potential for mild steel corrosion in 1 M HCl without the inhibitor (the blank system). This was attributed to dissolution of air oxide film on the mild steel surface [16,17]. A later increase in potential for this system after 50 s may be due to the formation of insoluble iron (III) oxide [16] on the mild steel surface leading to a more passive state of the steel. Initial increase in potential was observed in the presence of inhibitors followed by a sharp decrease after 75 s.

The nature of the OCP in the presence of SB was different from that of the blank. This is due to differences in surface activities on the steel in the absence and presence of the inhibitor. The OCP values for the inhibited systems were generally more positive than that of the uninhibited blank system. This can be attributed to the formation of protective film of the SB inhibitor on the steel surface [18] and suggests the inhibition of anodic dissolution of the steel by the SB under open circuit conditions [19]. Also, the OCP values increase with increase in inhibitor concentrations. This may be due to the increased number of inhibitor molecules in the protective layer formed on steel surface leading to thicker protective films. The OCP profile at 50 ppm of SB though reaches plateau at almost the same potential as the uninhibited 1 M HCl system, the 50 ppm SB containing electrochemical system obviously has a more positive potential on the average compared to the uninhibited system.

#### 2.2.2. Potentiodynamic Polarization Measurements

**Figure 2:**
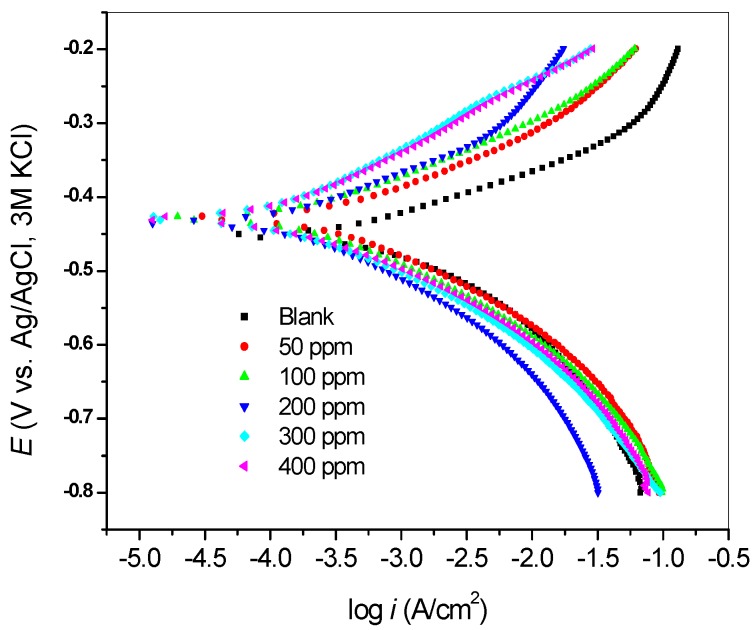
Polarization curves for the corrosion of mild steel in 1 M HCl with and without various concentrations of SB.

The polarization measurements were carried out on mild steel electrode immersed in 1 M HCl with and without various concentrations of SB after 30 min of immersion. Polarization curves were obtained as the plots of potential against logarithm of current density as presented in Figure 2. Electrochemical kinetic parameters including the corrosion current density (*i_corr_*), corrosion potential (*E_corr_*), anodic Tafel slope (*β_a_*), cathodic Tafel slope (*β_b_*) and percentage inhibition efficiency (*%IE*) were determined by linear extrapolations of Tafel lines within the straight-line regions of the polarization curves. These curves exhibit a shift to lower current density in the presence of inhibitor, which implies that SB reduces the rate of mild steel corrosion in 1 M HCl. The polarization curves are shifted to more positive (noble) values of *E_corr_* in the presence of SB. This suggests the formation of protective layer of SB on the steel surface. This is also in agreement with the observation during the OCP monitoring. Table 2 shows the kinetic parameters obtained after the linear Tafel fitting. An inhibitor can be regarded as anodic or cathodic type inhibitor if the shift in *E_corr_* value is greater than 85 mV [20]. As shown in Table 2, the maximum shift in *E_corr_* value in this study was 24 mV, which suggests that SB is a mixed-type inhibitor. That is, it reduces the rate of anodic reaction comprising the mild steel oxidation as well as cathodic reaction, which is hydrogen gas evolution. A closer look at the polarization curves (Figure 2), however, reveals that the anodic inhibiting effect is more pronounced in 1 M HCl, especially between 50 and 300 ppm, while at 400 ppm the mix-type inhibition characteristics is more obvious. This fact is also reflected in the relative magnitudes of the differences between the *β*a of the inhibitor containing and blank systems as well as the corresponding differences in the *β_c_* values as shown in Table 2. A maximum difference of 49 mV/dec was observed between the *β_a_* values in the absence and presence of inhibitor. Such a slight change in the values of *β*a and *β_c_* upon addition of the inhibitors when compared with the blank suggests that SB adsorbed onto the metal surface and inhibit the corrosion rate without changing the mechanism of the mild steel corrosion in hydrochloric acid [21].

The percentage inhibition efficiency (*%IE_P_*) was calculated at different concentrations of SB according to the equation:

(1) $$\%{\mathrm{IE}}_{\text{P}}=\left(\frac{{\text{i}}_{\mathrm{corr}}^{0}-{\text{i}}_{\mathrm{corr}}}{{\text{i}}_{\mathrm{corr}}}\right)\times 100$$

where *i^0^_corr_* and *i_corr_* are the corrosion current density with and without various concentrations of SB, respectively. The values of *%IE_P_* at various concentrations of SB are presented in Table 2. It is clear that *%IE_P_* increases with increase in concentration of SB with the highest value of 80.6% obtained at 400 ppm of the inhibitor.

**Table 2:** Electrochemical kinetic parameters from potentiodynamic polarization experiment.

Inhibitor Concentration (ppm)	−*E* (mV)	*i_corr_* (µA/cm^2^)	*β_a_* (mV/dec)	*β_c_* (mV/dec)	*R_p_* (Ω)	*%IE*
Blank	452	529.7	58	102	4.832	-
50	428	310.9	71	94	9.34	41.31
100	428	214.6	72	97	14.16	59.49
200	434	189.6	82	108	20.41	64.21
300	428	172.0	107	101	27.5	67.53
400	429	102.7	86	73	26.44	80.61

#### 2.2.3. Electrochemical Impedance Spectroscopy (Eis) Measurements

**Figure 3:**
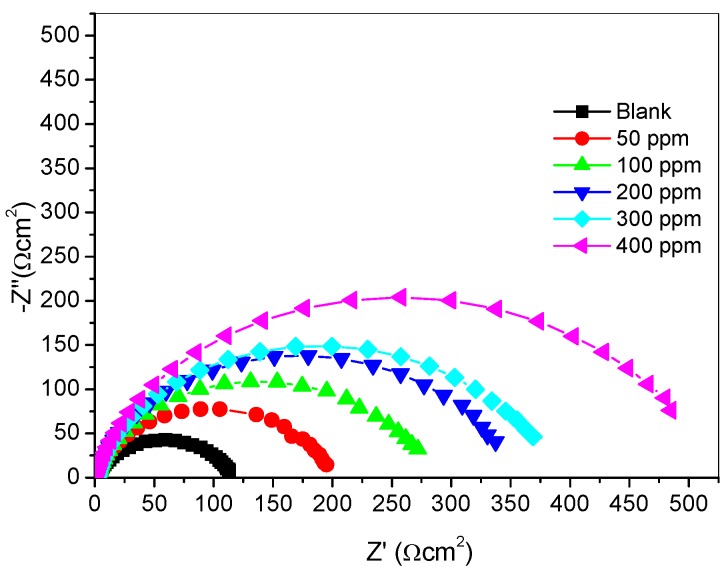
Nyquist plots for mild steel corrosion in 1 M HCl with and without different concentrations of SB.

The Nyquist plots obtained from the EIS studies are presented in Figure 3. The Nyquist plots in Figure 3 show a single depressed capacitive arc over the frequency range studied. This is an indication that the dissolution of mild steel in 1 M HCl is controlled by a single charge transfer process [22]. It was observed that the diameter of the semicircles in the Nyquist plots increases with increase in concentration of the SB inhibitor. Impedance data fitted properly with Randles equivalent circuit of the form *R_s_*(*R_ct_Q*), which consists of solution resistance (*R_s_*), in series with the parallel combination of the constant phase element (CPE), denoted as *Q,* and a charge-transfer resistance (*R_ct_*). Due to non-ideal capacitive behavior of the electrode/electrolyte systems investigated in the present study, the CPE was introduced in order to obtain a good agreement between experimental and simulated EIS data. The impedance (Z) of the CPE is defined as:

(2) $$Z_{CPE}=Q^{-1}{\left(j\omega \right)}^{-n}$$

where *Q* is the CPE constant (in Ω^−1^S^n^cm^−2^); *j*^2^ = −1 is the imaginary number; *ω* is the angular frequency (in rads^−1^); and *n* is a CPE exponent, which can be used as a gauge of the heterogeneity or roughness of the surface.

The CPE can represent resistance (n = 0, Q = R), capacitance (n = 1, Q = C), inductance (n = −1, Q = L), or Warburg impedance (n = 0.5, Q = W). Thus, the closer the value of n to unity, the better the capacitive behavior of Q. The electrochemical kinetic parameters obtained from the fitting of impedance data are presented in Table 3. Since the CPE exponent, n, is close to 1 for the studied system, Q can be assumed to have some capacitive features and referred to as the double-layer capacitance. The percentage inhibition efficiency (*%IE_EIS_*) was calculated by using the equation:

(3) $$\%IE_{EIS}=\left(\frac{R_{ct}-R_{ct}^{0}}{R_{ct}}\right)\times 100$$

where $R_{ct}^{0}$ and *R_ct_* are the charge transfer resistances with and without various concentrations of SB inhibitor, respectively. As shown in Table 3, *%IE_EIS_* increases with increase in concentration of SB. This confirms the inhibition potency of SB against mild steel corrosion in 1 M HCl. There is an increase in the values of *R_ct_* as the concentration of SB increases. This is attributed to an increase in the interface between the metal surface and the aggressive solution due to increase in the area of the adsorption film formed on the metal surface. According to the Helmholtz model [23], Q is expressed as:

(4) $$Q=\frac{\varepsilon^{0}\varepsilon }{d}S$$

where *ε^0^* is the permittivity of the vacuum; ε is the dielectric constant of the medium; *d* is the thickness of the film and S is the surface area of the electrode. The decrease in values of *Q* as the concentration of SB increases may be due to an increase in the area of the adsorption film, which corresponds to the decrease in the exposed electrode surface area (*S*), or an increase in the thickness of the adsorbed protective layer (*d*) or a decrease in the medium dielectric constant (*ε*). One or more of these result in the observed decrease in the values of *Q* in accordance to Equation (4) above.

**Figure 4:**
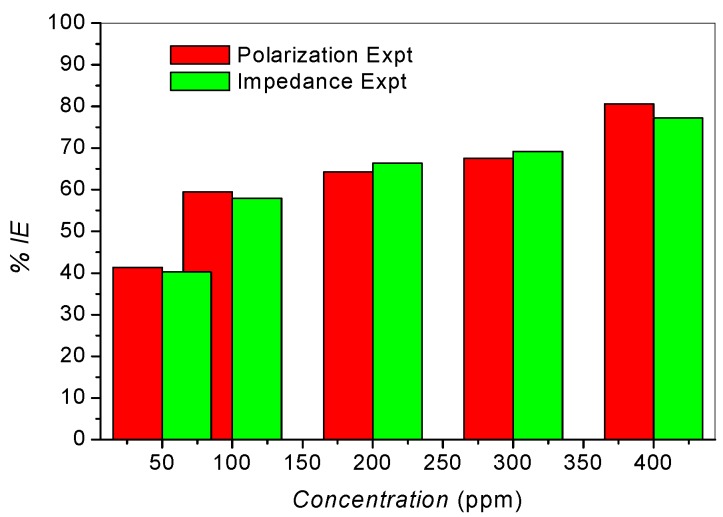
Variation of percentage inhibition efficiency (%*IE*) with concentration of SB for both polarization and impedance experiments.

The variations of percentage inhibition efficiency with concentrations of SB are plotted in Figure 4. There is a good agreement between the values obtained for the percentage inhibition efficiency from the polarization and impedance techniques. It is worthy of mention that the studied compound (SB) when compared with some previously reported organic inhibitors exhibits similar or relatively better inhibition performance for mild steel corrosion in 1 M HCl [24,25,26].

**Table 3:** Fitted parameters from electrochemical impedance spectroscopy.

Inhibitor Concentration (ppm)	*R_s_* (Ωcm^−2^)	*R_ct_* (Ωcm^−2^)	*Q_1_* (Y_0_) (µFcm^−2^)	*n*	*% IE_EIS_*
Blank	1.947	115.30	145.00	0.8027	-
50	2.570	193.00	85.58	0.8756	40.26
100	1.808	274.10	82.21	0.8651	57.94
200	1.937	343.00	71.67	0.8736	66.38
300	1.754	374.00	64.57	0.8707	69.17
400	1.504	506.00	61.56	0.8718	77.21

### 2.3. Adsorption Isotherms

**Figure 5:**
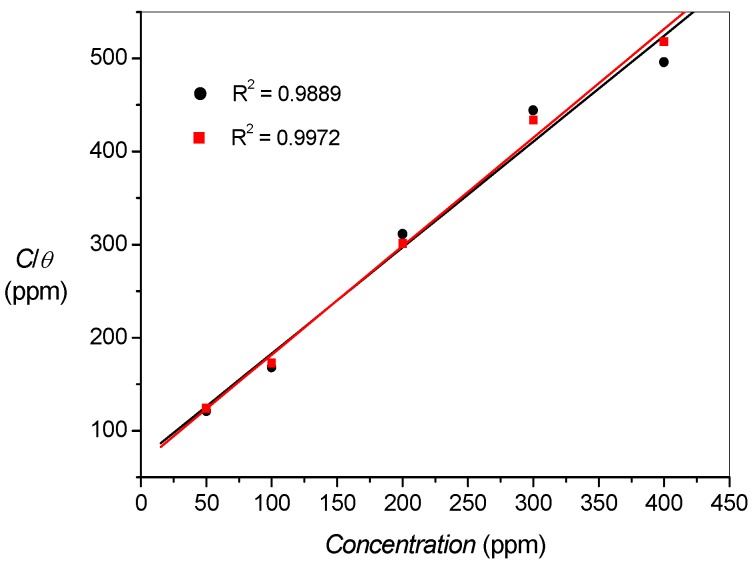
Langmuir adsorption isotherms of SB adsorption on mild steel surface from (▪) polarization and (●) impedance experiments.

Important information on the adsorption behavior of an inhibitor on a metal surface can be obtained by fitting the experimental data into appropriate adsorption isotherms. The adsorption of inhibitor on metal/solution interface may occur through the displacement of water molecules by the inhibitor molecules [27] in accordance to the reaction equation:

Inh_(sol)_ + *x*H_2_O_(ads)_ ↔ Inh_(ads)_ + *x*H_2_O_(sol)_ (5)

where, *x*, the mole ratio, is the number of water molecules replaced by one molecule of organic inhibitor. The surface coverage, *θ*, was calculated from the percentage inhibition efficiency (*θ* = *%IE*/100), obtained from both polarization and impedance measurements. The experimental data obtained fitted well with the Langmuir adsorption isotherm represented by the equation:

(6) $$\frac{C_{inh}}{\theta }=\frac{1}{K_{ads}}+C_{inh}$$

where *C_inh_* is the concentration of the inhibitor and *K_ads_* is the equilibrium adsorption constant. The plots of *C_inh_/θ vs C_inh_* (Figure 5) gave straight lines with strong linear correlation coefficients.

The change in free energy of adsorption (*∆G_ads_*) was calculated from the relation:

(7) $$\text{Δ}G_{ads}=-RT\ln\left(55.5K_{ads}\right)$$

where *R* is gas constant; *T* is absolute temperature and the constant 55.5 is the molar concentration of water. The values of *K_ads_* and *∆G_ads_* are presented in Table 4. The negative value of *∆G_ads_* implies that the adsorption process is spontaneous. The magnitude of *∆G_ads_* is usually used to predict the nature of adsorption, whether it is physisorption or chemisorption. A value of *∆G_ads_* around −20 kJ/mol or less negative has been attributed to electrostatic interactions between the charged inhibitor molecules and the charged metal surface (physisorption), while values around −40 kJ/mol, or larger negative values, involve charge sharing or charge transfer from organic molecules to the metal surface to form coordinate bond (chemisorption) [28]. The magnitudes of *∆G_ads_* obtained in the present study suggest that the adsorption of SB on mild steel surface is a combination of both physisorption and chemisorption processes.

**Table 4:** Adsorption constant and change in free energy of adsorption of SB on mild steel surface from polarization and impedance experiments at 303 K.

Title	*K_ads_* (×10^−3^)	− *∆G_ads_* (kJ/mol)
Polarization Expt.	6.47	31.71
Impedance Expt.	6.89	31.86

### 2.4. Ultraviolet-Visible and Raman Spectroscopic Studies

**Figure 6:**
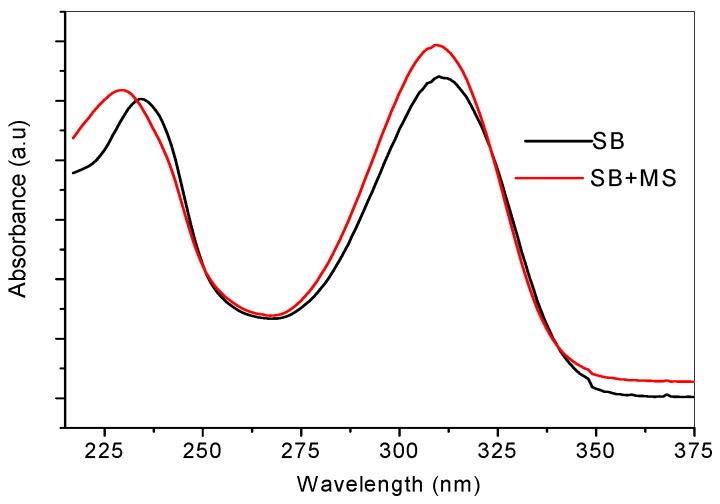
UV-Vis spectra of pure SB solution (black, SB) and SB solution after mild steel immersion (red, SB+MS).

UV-vis and Raman spectroscopic analyses were carried out on pure SB and the resulting solution of SB after five days of mild steel immersion. Comparison of the spectroscopic features of pure SB and SB with mild steel can provide information about possible formation of Fe/SB complex. The UV-vis spectrum of SB solution (Figure 6) shows two absorption peaks, at 310 nm and 234 nm, corresponding to n→π* transition, π→π* transition and intermolecular charge transfer. After mild steel immersion, the maximum absorption peak seems to be unaffected as it re-appeared at 309 nm. However, there is a slight blue shift of the peak at 234 nm to 229 nm. This may be attributed to the formation of coordinate bonds between Fe and SB.

**Figure 7:**
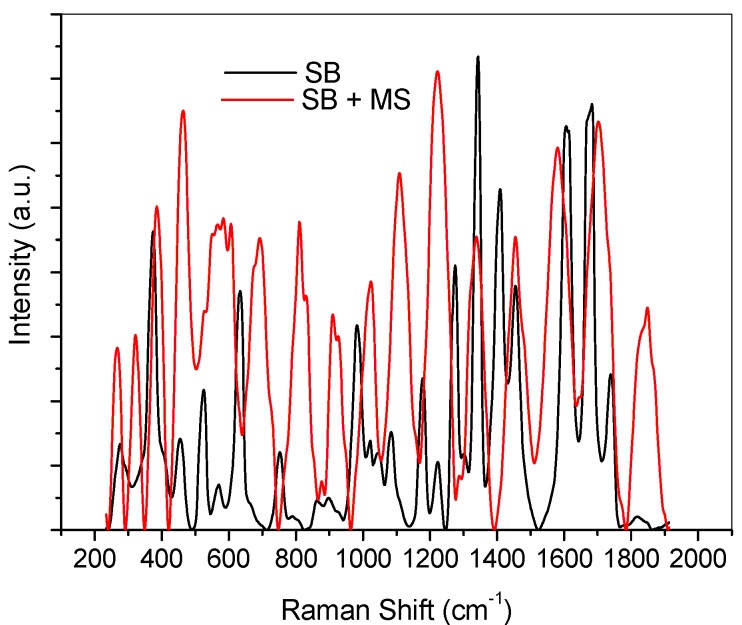
Raman spectra of pure SB solution (black, SB) and SB solution after mild steel immersion (red, SB + MS).

The Raman spectroscopic analysis of possible formation Fe/SB complex was carried out by investigating the change in prominent Raman bands between 200 cm^−1^ and 2000 cm^−1^ after mild steel immersion in SB solution. As shown in Figure 7, in the pure SB, a strong band at 371 cm^−1^ is assigned to aliphatic CC chains in the molecule, while a medium peak, characteristic of C-O-C band appears at 978 cm^−1^, coupled with its asymmetric band at 1074 cm^−1^. Aromatic CC bands of medium intensity appear at 1173 cm^−1^ and 1451 cm^−1^. Two strong bands appear at 1602 cm^−1^ and 1674 cm^−1^ which may be due to C=N functional group. The band at 1732 cm^−1^ is of medium intensity and it is attributed to C=O. After mild steel immersion, more bands appear in the region below 500 cm^−1^. The strong bands at 382 cm^−1^ and 458 cm^−1^ are of special interest and can be assigned to Fe-O, suggesting bond formation between SB and Fe through the sp^2^ oxygen. Other bands have also shifted correspondingly after mild steel immersion in SB. Most of the bands shifted to higher wavenumbers after mild steel immersion. This includes the bands for C=N functional groups.

### 2.5. Quantum Chemical Calculations

**Figure 8:**
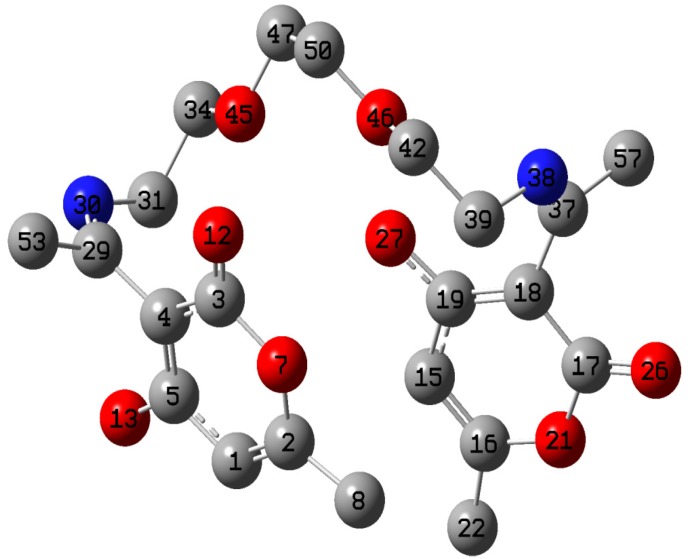
Optimized structure of SB showing only non-hydrogen atoms with atomic numbering.

The gas phase optimized structure of SB with atom labels is shown in Figure 8. SB is expected to be a symmetrical molecule but the optimized structure shows that there is extension of π-conjugation to the O27 atom of the –OH group, whereas this is not observed on the similar oxygen atom O13 of the –OH group on the other end of the molecule. This non-uniform distribution of electron density makes the molecule to be unsymmetrical. Relevant quantum chemical parameters of the optimized structure are listed in Table 5. Adsorption of an inhibitor on a metal surface is often explained based on donor-acceptor phenomenon between the inhibitor and metal atom. The energy of the highest occupied molecular orbitals (*E_HOMO_*) is associated with the tendency of an inhibitor molecule to donate its least stable electron(s) to the appropriate vacant orbitals of the metal atom. On the other hand, the energy of the lowest unoccupied molecular orbitals (*E_LUMO_*) informs the tendency of the inhibitor molecule to accept charges from the metal atom towards back-bonding. The higher the *E_HOMO_*, the higher the possibility of forward donation of charges to the metal, and the lower the *E_LUMO_*, the better the chances of back-donation of charges. The results in Table 5 show that the values of *E_HOMO_*, *E_LUMO_* and energy gap (*∆E_LUMO-HOMO_*), the ionization potential (I = −*E_HOMO_*) and the electron affinity (A = −*E_LUMO_*) of SB are within the range of values that have been reported for some other Schiff bases that had also been adjudged good corrosion inhibitors [29]. SB has a reasonably high dipole moment, which suggests that it has the potential to interact with metal atom or ions in aqueous system. The results of the chemical potential (*µ*), hardness (*η*) and electrophilicity (*ω*) as shown in Table 5 reveal that SB has a potential to react with Fe in the mild steel thereby protecting the steel surface against corrosion. The value obtained for fraction of electrons transferred (∆*N*) from the inhibitor molecule to the metal surface confirms the possibility of charge transfers from SB to the mild steel.

**Table 5:** Some gas-phase quantum chemical parameters of SB calculated at B3LYP/6-31G(d) level.

Parameter	Value
Total energy (au)	−1566.4527
*E_HOMO_* (eV)	−5.99
*E_LUMO_* (eV)	−1.46
∆*E_LUMO-HOMO_*	4.53
*I*	5.99
*A*	1.46
Dipole moment (Debye)	5.69
Chemical potential, *µ* (eV)	−3.72
Hardness, *η* (eV)	2.26
Electrophilicity, ω (eV)	3.06
∆*N*	0.72
∆*E* (eV)	-0.57

**Figure 9:**
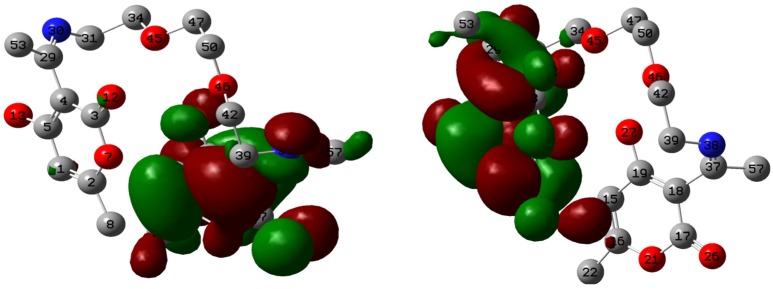
HOMO and LUMO surfaces of SB at an isosurface value of 0.02.

**Figure 10:**
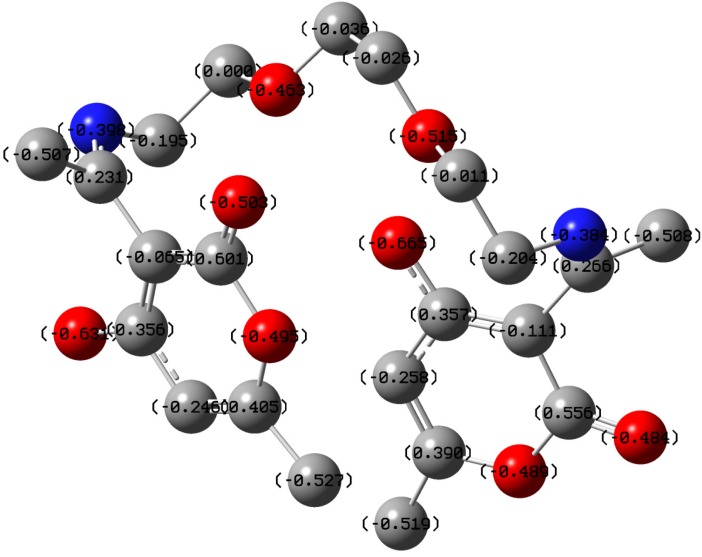
Mulliken atomic charges of atoms in the optimized structure of SB.

The change in energy (∆*E*) following charge transfer implies that the charge transfer from SB to mild steel followed by back donation from mild steel to the inhibitor is energetically favorable. The HOMO and LUMO graphic surfaces (Figure 9) are distributed around the unsaturated ring of the dehydroacetic acid with the HOMO surface being around the ring with the unsaturated –OH group and the LUMO surface on the ring that has no π-conjugation to the –OH. The Mulliken atomic charges of SB are displayed on the atoms in the optimized structure (Figure 10). There are quite a number of atoms with negative Mulliken charges, which implies that SB has potential atoms that can interact with relatively positive centers on the mild steel surface.

The Fukui functions (*f*(r)) are often used as indices of local reactivity to analyze the active atomic sites in inhibitor molecules [30,31]. The Fukui functions (*f* (r)) measure the change in the electron density of an N electron system upon addition (*f*^+^(r)) or removal (*f*^−^(r)) of an electron [32]. Atom condensed Fukui functions using the Mulliken population analysis (MPA) and the finite difference (FD) approximations approach introduced by Yang and Mortier [33] were calculated using the equations:

(8) $$f_{k}^{+}=\rho_{k(N+1)}(r)-\rho_{k(N)}(r)$$

(9) $$f_{k}^{-}=\rho_{k(N)}(r)-\rho_{k(N-1)}(r)$$

where $\rho_{k(N+1)}$, $\rho_{k(N)}$ and $\rho_{k(N-1)}$ are the electron densities of the *(N+1)-*, *N-* and *(N-1)-* electron systems, respectively, and approximated by Mulliken gross charges; *f*^+^ and *f*^−^ are the Fukui indices condensed on atom *k* and measure its electrophilic and nucleophilic tendencies, respectively. The Fukui indices are listed in Table 6 for selected non-hydrogen atoms in SB. The atomic labels employed in Table 6 are the same as those displayed in Figure 8. The most susceptible sites for nucleophilic attacks are O45, C31, C47 and C50, while the most susceptible sites for electrophilic attack are C39, O45, C50, C47 and C31 in that order.

**Table 6:** Fukui indices for non-hydrogen selected atoms in SB molecule.

Atom	*f^+^*	Atom	*f^−^*
C1	0.0013	C8	0.0022
C8	0.0098	C22	0.0085
C15	0.0104	C29	0.0054
C22	0.0072	C31	0.0102
C31	0.0182	C34	0.0097
C34	0.0076	C37	0.0005
C39	0.0040	C39	0.0163
C42	0.0081	C42	0.0077
O45	0.0215	O45	0.0135
O46	0.0078	O46	0.0088
C47	0.0127	C47	0.0114
C50	0.0102	C50	0.0136
C53	0.0076	C53	0.0042
C57	0.0052	C57	0.0072

## 3. Experimental Section

### 3.1. Materials

All reagents and solvents were of analytical grade and were used without further purification. The experiments were performed on mild steel samples with the chemical composition (wt %) C = 0.17, Mn = 0.46, Si = 0.26, S = 0.017, P = 0.019, and balance Fe. For all electrochemical studies, mild steel coupons were cut into 1 cm × 1 cm dimensions and embedded in a Teflon holder using epoxy resin, exposing only 1 cm^2^ surface area. Prior to each measurement, mild steel surface was mechanically abraded on Struers MD PianoTM 220 (size: 200 dia) mounted on Struers LaboPol-1 machine to remove traces of epoxy resin from the surface. The surface was then polished with SiC papers of various grit sizes ranging from 600 to 1200 to achieve a finely ground surface then washed with water followed by acetone and then water again, and finally wiped with clean tissue paper and air-dried. Mild steel specimens were used immediately after surface preparation.

### 3.2. Chemicals and Instrumentation

3-acetyl-4-hydroxy-6-methyl-(2H) pyran-2-one and 2,2'-(ethylenedioxy)diethylamine were used as supplied by Fluka. Elemental analyses of C, H and N were performed by using Carlo Erba Elemental analyzer EA 1108. Melting point was taken in open capillaries on a melting point apparatus model no 125. IR spectra were recorded on a Perkin Elmer Spectrum 100. 1H and 13C NMR spectra were obtained from a Bruker AV 500 MHz for 1H and 125 MHz for 13C using a 5 mm Quadra Nuclei Probe (QNP). UV-Vis spectra were recorded on Cary 300 UV-Vis by Agilent Technologies. Raman spectra were obtained from Xplora Raman spectrometer from Horiba Scientific.

### 3.3. Synthesis of 3-acetyl-4-hydroxy-6-methyl-(2H) pyran-2-one Schiff base (SB)

A solution of 3-acetyl-4-hydroxy-6-methyl-(2H) pyran-2-one (3.36 g, 0.02 mol) in 20 mL ethanol was mixed with a solution of 2,2'-(ethylendioxy) diethylamine in 20 mL ethanol. The equation of reaction is as shown in Equation (10):

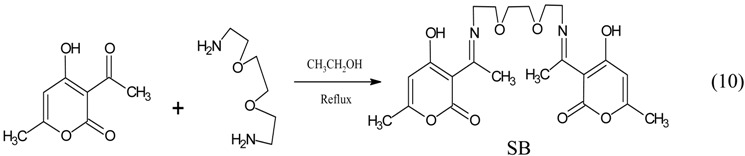
(10)

The mixture was refluxed for 3 h, and the resulting solution was chilled to −10 °C to obtain a whitish product which was filtered, dried and recrystallized in water.

### 3.4. Electrochemical Measurements

All electrochemical measurements were carried out on the Autolab PGSTAT 302N obtained from Metrohm, equipped with a three-electrode system. Ag/AgCl with 3 M KCl was used as the reference electrode, while platinum rod was used as the counter electrode.

The system was allowed to reach the steady open circuit potential (OCP) before each electrochemical measurement. The OCP measurements were carried out for 30 min in the aggressive solutions with and without various concentrations of SB. The systems were confirmed to have reached OCP before 30 min with less than ±10 mV change in potential. The potentiodynamic polarization tests were performed after 30 min of mild steel immersion in the aggressive solutions by sweeping the potential between –200 mV and –800 mV *versus* Ag/AgCl, 3 M KCl reference electrode potential at the scan rate of 1 mV/s. Electrochemical impedance spectroscopy measurements were carried out after 30 min of mild steel immersion in the aggressive solution with and without various concentrations of the inhibitor. The electrochemical impedance spectroscopy measurements were conducted at the OCP by analyzing the frequency response of the electrochemical system in the range of 100 kHz to 1 Hz at 5 mV amplitude. All electrochemical experiments were conducted under unstirred conditions at 303 K.

### 3.5. Quantum Chemical Calculations

All quantum chemical calculations were carried out using the DFT method involving the Becke 3-Parameter exchange functional together with the Lee-Yang-Parr correlation functional (B3LYP) [34,35] and 6-31G(d) basis set. SB was modeled with Gaussview 5.0 software to obtain the initial geometries. Gas phase geometry optimization was carried out using Gaussian 09W D.01 software [36].

## 4. Conclusions

The new Schiff base (SB) synthesized from the condensation of 3-acetyl-4-hydroxy-6-methyl-(2H)pyran-2-one and 2,2'-(ethylenedioxy)diethylamine showed corrosion inhibition potency for the protection of mild steel in 1 M HCl, as confirmed by potentiodynamic polarization and electrochemical impedance spectroscopy experiments. SB was found to be a mixed-type inhibitor and its inhibition property was associated with its spontaneous adsorption onto mild steel surface via both physisorption and chemisorption. The experimental data fitted Langmuir adsorption isotherm. UV-vis and Raman spectroscopic analyses revealed the possibilities of chemical interactions and bond formation between mild steel and SB. Quantum chemical calculations suggested the possible sites for nucleophilic and electrophilic attack on SB.
